# Correction: Structure-based design of an aromatic helical foldamer–protein interface

**DOI:** 10.1039/d6sc90042a

**Published:** 2026-02-17

**Authors:** Lingfei Wang, Céline Douat, Johannes Sigl, Post Sai Reddy, Lucile Fischer, Béatrice Langlois d'Estaintot, Zhiwei Liu, Vojislava Pophristic, Yuwei Yang, Yingkai Zhang, Ivan Huc

**Affiliations:** a Department Pharmazie, Ludwig-Maximilians-Universität München Butenandtstr. 5–13 81377 München Germany ivan.huc@cup.lmu.de; b CNRS, Bordeaux INP, CBMN, UMR5248, Univ. Bordeaux F-33600 Pessac France; c Department of Chemistry & Biochemistry, Rowan University 201 Mullica Hill Road 08028 Glassboro New Jersey USA; d Department of Chemistry, New York University 10003 New York New York USA

## Abstract

Correction for ‘Structure-based design of an aromatic helical foldamer–protein interface’ by Lingfei Wang *et al.*, *Chem. Sci.*, 2025, **16**, 12385–12396, https://doi.org/10.1039/D5SC01826A.

The authors regret that a PDB accession number was incorrect in the Data availability section of the original article. The Data availability section for this article with the correct number (9HGP) is shown below. The error was also corrected in two instances in the supplementary information document.

Updated supplementary information is available alongside the original article, https://doi.org/10.1039/D5SC01826A, and the new Data availability statement is as follows:

**Data availability**

The data supporting this article have been included as part of the supplementary information (SI). Crystallographic data for **2**·HCAII, **3**·HCAII, **16**·HCAII and **20**·HCAII have been deposited at the Protein Data bank under accession numbers 9GAM, 9GAK, 9GAJ, 9HGP, respectively, and can be obtained from https://doi.org/10.2210/pdbXXXX/pdb where XXXX is the accession number.

The Royal Society of Chemistry apologises for these errors and any consequent inconvenience to authors and readers.

